# Post-coital traumatic haemorrhage as cause of death

**DOI:** 10.1007/s12024-025-01143-z

**Published:** 2026-02-09

**Authors:** Anton Mittendorf, Carl Winskog

**Affiliations:** 1 Swedish National Board of Forensic Medicine, Gothenburg Unit, Box 408, 405 30 Göteborg, Sweden; 2 Department of Oncology-Pathology, Karolinska Institute, Stockholm, Sweden

**Keywords:** Sexual intercourse, Traumatology, Cause of death, Forensic Medicine

## Abstract

This case report describes the death of a 40-year-old female who was found dead in her home after a vaginal bleeding. Her sexual partner described in police hearings that the bleeding started during coitus the previous day.

## Introduction

Minor injuries, such as bruising or laceration of the vaginal mucosa after sexual intercourse is a well described finding and can be seen both after consensual and non- consensual sex [1–3]. Major injuries, while uncommon, are not unheard of [4] and in the fornix area, major injuries during sexual intercourse have been described previously, including perforation [4] and evisceration [5]. Causes and risk factors of vaginal injuries include vaginal delivery, trauma, tumour, congenital anomalies of vaginal development and menopause [6]. The vaginal fornix is located in the area where there is blood supply from both the uterine artery and the vaginal artery [7] and adjacent to the outer vascular zone of the cervix, containing the larger arteries and veins of the anatomical structure [8]. During intercourse, there is a significant lengthwise stretching of the vaginal wall, with the point of impact for the penile penetration being around the fornix [9]. Histologically, the mucosa of the fornix and ectocervix is lined with stratified squamous epithelial cells atop a stromal cell layer and harbours tissue-resident immune cells, such as memory CD4 + and CD8 + T-cells [10].

## Case presentation

A 40-year-old female who was found dead in her home by the police. The body was lying face down naked on the bedroom floor next to the bed. Bruises were noted on the body and there were bloody sheets and a blood-soaked towel on the bed. Clotted blood, a blood-soaked tampon and blood stains suggesting an impact angle of near 90 degrees, partially with a low impact droplet-in-droplet pattern with satellite splashes [11], was seen in the living room and bath room. Blood had also soaked into the sofa-bed in the living room.

The police had been alerted by the emergency assistance centre, who had been contacted by a man who had had sexual intercourse with the deceased the previous night. He was taken in for questioning and told the police that he and the deceased woman had had consensual vaginal sex the night before. The sexual positions involved were not described in the police report. The two had known each other for several years before but were not in an official relationship. He had not thought that she seemed intoxicated at the time but she did drink wine while he was with her. He noticed that she had bruises, but she had told him that they were from playing football. During the intercourse they noticed a vaginal bleeding, which would not stop. No mention was made as to whether she complained of pain. He told the police that he had been worried and suggested that they call an ambulance, which she refused. He did not spend the night with the deceased and could not get in contact with her the following morning or during the day and called emergency services when he started to get worried. An ambulance was present, the staff noting that resuscitation attempts were futile but the police report does not explicitly state whether attempts were made initially or not.

According to the police report, the deceased woman had a history of alcohol abuse and a DUI. Disulfiram, acamprosate, propiomazine and zolpidem was found in her home. It was not known whether she had undergone menopausal changes.

## Clinical findings

The autopsy was performed at the Gothenburg Forensic Pathology unit of the Swedish National Board of Forensic Medicine two days after the deceased was found and three days after she was last seen alive according to the police report. No PMCT was performed.

Livor mortis was noted as pale and sparse in the autopsy protocol.

The external examination revealed a small laceration on the right side of the lower lip together with four minor injuries to the oral mucosa of the right cheek, suggesting a self-inflicted bite mark.

Bruises of varying ages were seen on both arms, on the back, on the belly, on the right side of the gluteal region and on both legs. Some of the bruises, on the back side of the upper arms and on the lateral side of the left thigh, suggested finger marks from gripping.

Minor abrasions were seen on the arms.

Blood was seen in the vaginal opening, but no injuries were seen on the outer genitalia or in the anal region on external examination.

No signs of resuscitation attempts were seen.

## Autopsy findings

The lungs, the liver and the kidneys were noted as being pale and the lungs did yield only a small amount of fluid when pressed.

**Fig. 1:**
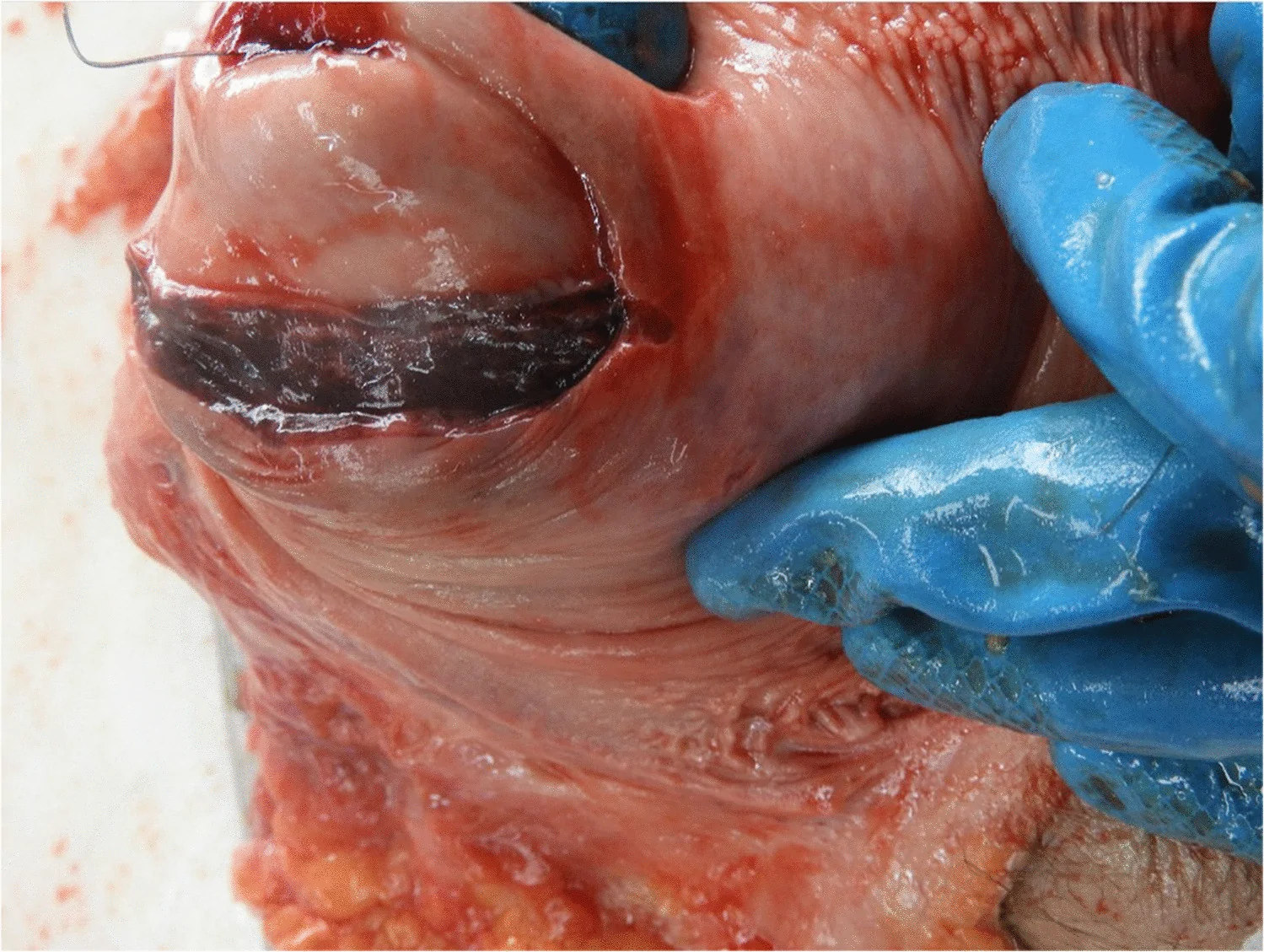
The laceration, located around 3 cm from the cervical canal

**Fig. 2:**
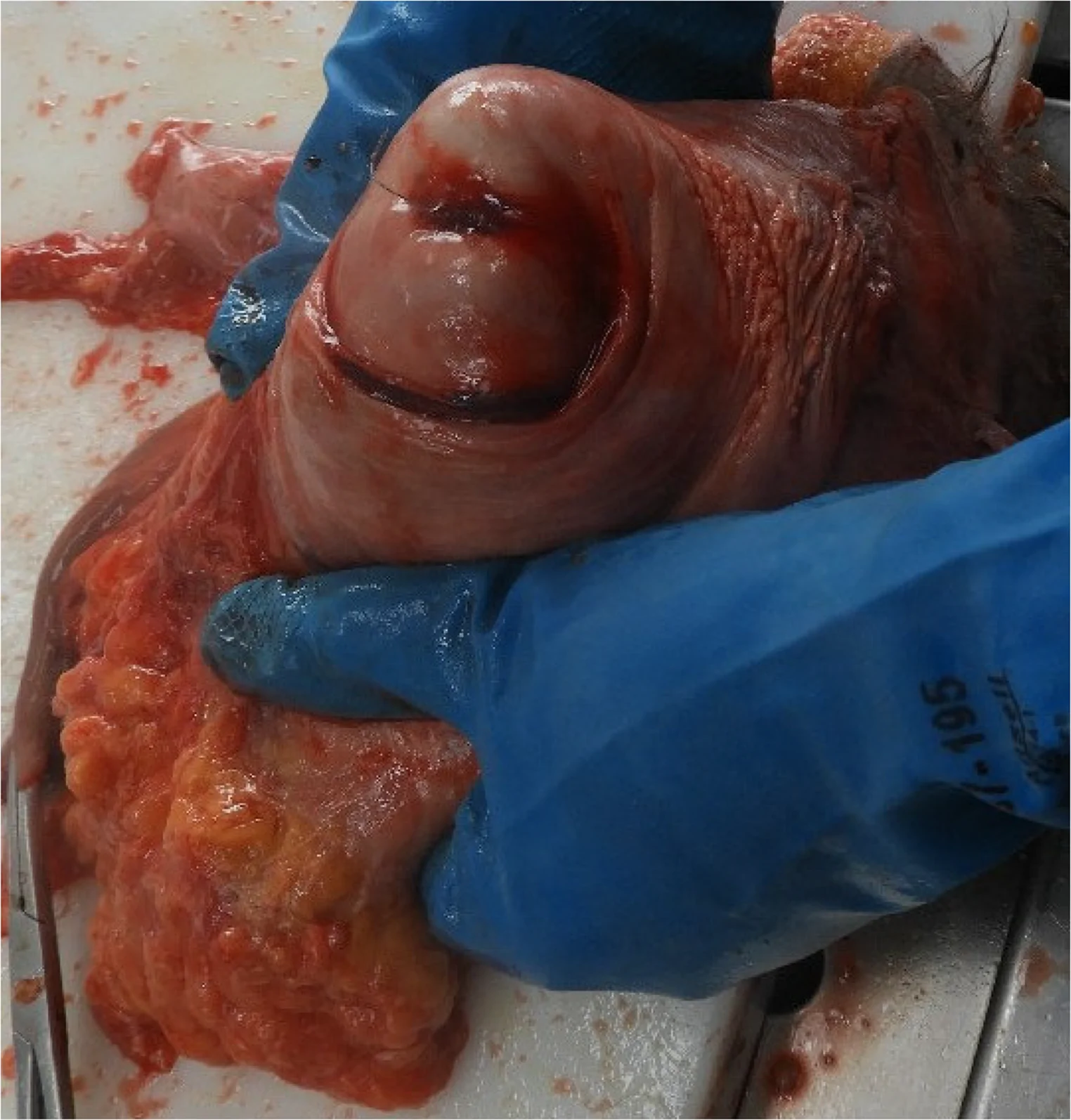
Overview

**Fig. 3:**
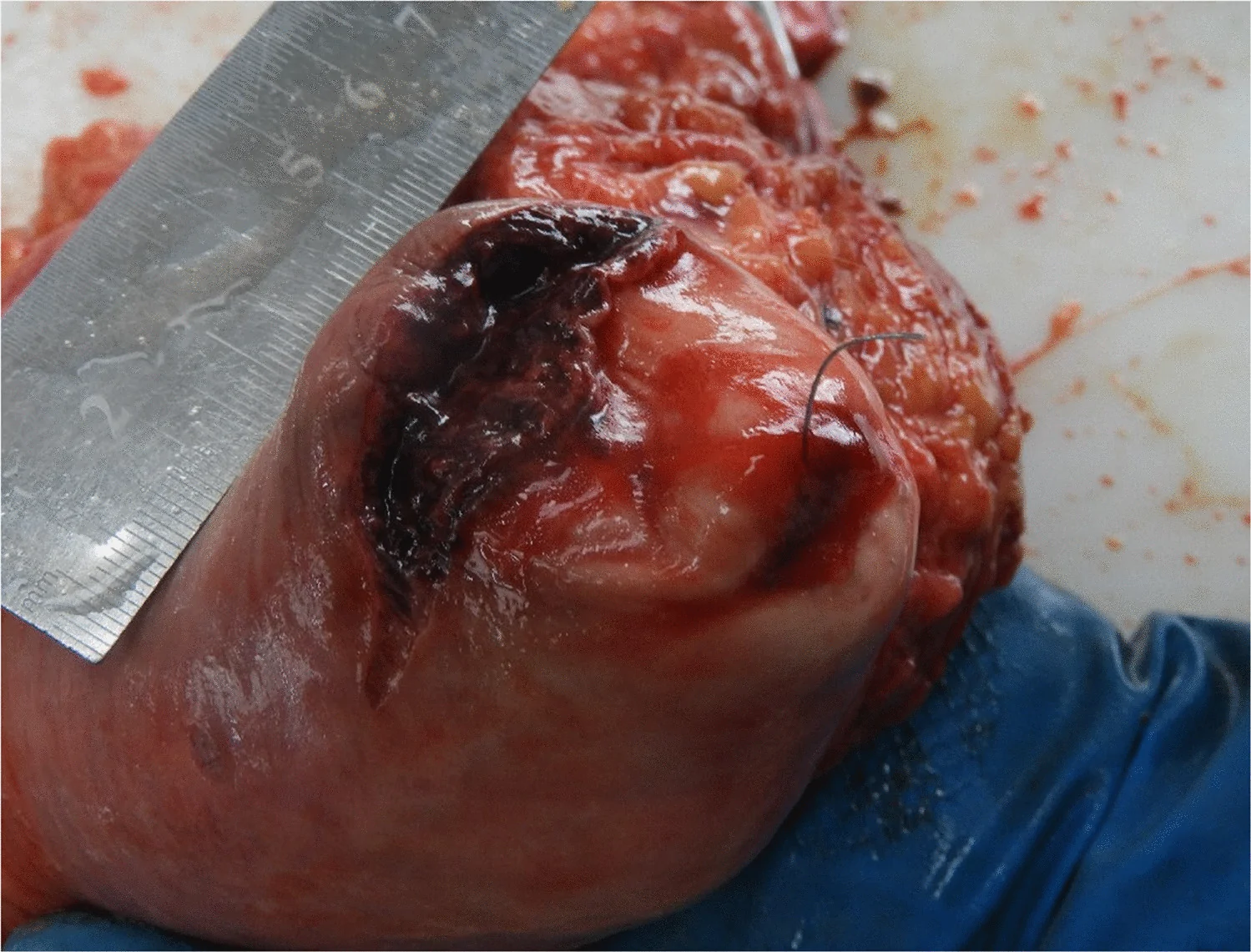
The laceration, with ruler (centimetres)

**Fig. 4:**
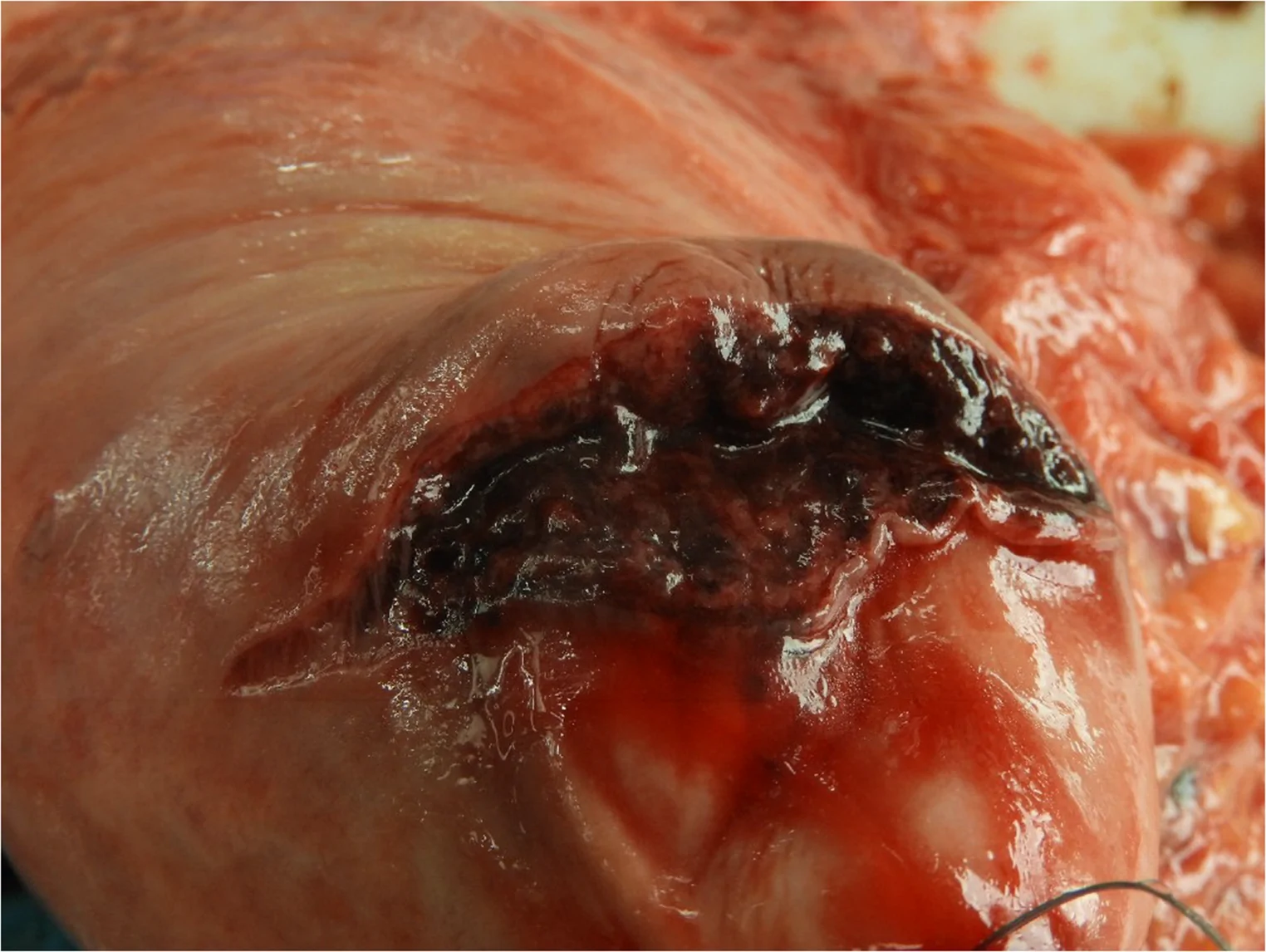
The exposed area of tissue in the laceration

During autopsy, a vulvectomy was performed. This showed a 5 × 1,5 cm laceration with a depth of approximatively 0,7 cm to the left of the cervix in the fornix area (Figs. 1 and 2). Partially congealed blood was seen next to the injury and the exposed tissue in the wound showed signs of partial coagulation (Figs. 3 and 4).

In the uterus an IUD was found. No other injuries were seen in the genital tract. No tumours or anatomical anomalies were seen.

A suspected steatosis of the liver was noted. No signs of resuscitation attempts were seen.

## Histological findings

Histological examinations of the vaginal mucosa in the injured area showed haemorrhage. Both neutrophils and lymphocytes were seen. The edges of the injured area showed necrotic tissue.

## Chemical analysis

Blood from the femoral vein and urine revealed elevated ethanol content (1.2 ‰ and 1.8 ‰, respectively), indicating alcohol intoxication. Acetone was found in femoral blood and urine (0.076 ‰ and 0.18 ‰) and β-hydroxybutyric acid was found in femoral blood in a high concentration (650 μg/g). Prometazine and sertraline was found in femoral blood in therapeutic concentrations. No other substances were detected.

## Conclusion

Exsanguination, air embolism and ketoacidosis were all considered as causes of death. Taking into account the amount of blood found on the scene and the cadaveric signs suggesting anaemia, the cause of death was determined to be external haemorrhage due to the vaginal injury but with alcohol-related ketoacidosis as a contributing factor [12].

The vaginal injury and some of the bruising was determined to having been most likely caused by another person, through vaginal penetration and gripping, respectively. There was no information that an object had been used during the sex act but the use of an object could neither be confirmed or ruled out.

As lymphocytes can be observed in healthy vaginal mucosa, the findings of such cell should not be interpreted as a sign of advanced wound healing progress. Murine models suggest that while leukocytes in general are present before injury in the vaginal mucosa, neutrophils are not, but can be observed 12 h after injury concurrent with a reduction in overall leukocyte numbers [13]. While human conditions should be extrapolated with care, this suggests that some hours elapsed between injury and time of death, as both neutrophils and other leukocyte cell lines were present.

As parts of the bruising was consistent with grip marks, coercion could not be ruled out. However, disinhibition due to inebriation could also be pondered as a contributing factor both to the bruising and the vaginal injury. Considering the lack of further findings supporting sexual violence as well as the histologically suggested time frame allowing for actions by the deceased after injury, it was deemed impossible to determine with any degree of certainty whether the penetration had been consensual or not. Thus, the manner of death was thus noted as undetermined.

This case highlights the sensitivity of the fornix during intercourse as well as the dangers of neglecting unexpected vaginal bleeding. In addition, it serves as a reminder of the increased risk of severe bleeding in the acidotic patient.
